# Should there be greater use of preprint servers for publishing reports of biomedical science?

**DOI:** 10.12688/f1000research.8229.1

**Published:** 2016-03-03

**Authors:** Iain Chalmers, Paul Glasziou

**Affiliations:** 1 James Lind Initiative, Oxford, UK; 2 REWARD Alliance, Bond University, Gold Coast, Australia

**Keywords:** preprint, publishing, peer review, journals, biomedicine

## Abstract

Vitek Tracz and Rebecca Lawrence declare the current journal publishing system to be broken beyond repair. They propose that it should be replaced by immediate publication followed by transparent peer review as the starting place for more open and efficient reporting of science. While supporting this general objective, we suggest that research is needed both to understand why biomedical scientists have been slow to take up preprint options, as well as to assess the relative merits of this and other alternatives to journal publishing.

## Editorial

Vitek Tracz (Chairman of F1000, which includes *F1000Research*) and Rebecca Lawrence (Managing Director of F1000) declare the current journal publishing system to be broken beyond repair ( Tracz & Lawrence, 2016). They propose much greater use of immediate publication of articles as the starting place for open, interactive dialogues between study authors and commentators on reports of research (whether or not commentators are designated formally as ‘peer reviewers’). Logically, over the course of one or more iterations, this interaction should promote the evolution of more ‘stable’, trustworthy and useful records of research. Furthermore, new technology provides a much more efficient system for dealing with submitted reports of research than that offered by most current journals using traditional forms of peer-review.

As Tracz and Lawrence recognise, although the use of preprints for reporting research was introduced by particle physicists decades ago, it has not attracted much tangible support from life scientists. Why is this; and what might be the downsides of their proposed preprint-led revolution?

Part of the explanation seems likely to reflect reasonable concern about the danger that preprints reporting flawed research may prompt unwarranted changes in clinical practice that harm patients. Tabor (2016), commenting on a recent call for a prepublication culture in clinical research like that established in some areas of physics ( Lauer *et al*., 2015), noted that “clinical studies of poor quality can harm patients who might start or stop therapy in response to faulty data, whereas little short-term harm would be expected from an unreviewed astronomy study”.

Reasonable people can identify with this concern about the potential dangers of an increased use of preprints in biomedicine. However, other reasons for the poor uptake of preprint opportunities seem likely to reflect less worthy, often perverse interests, reflecting financial or academic conflicts, or just acquiescence in sloppy science.

Despite their recognition of the increasingly acknowledged importance of collaboration, the model proposed by Tracz and Lawrence focuses on “individual researcher’s scientific output”, and they believe that the model they propose would work only if driven by authors “within a scientific framework that facilitates self-regulation”. This judgement ignores the abundant evidence that self-regulation by researchers cannot alone deal adequately with many of the systemic problems apart from journals which are leading to waste, bad science, bad ethics and harm to patients.

Although Tracz and Lawrence introduce their proposals with an allusion to “the need to remove the waste in the current system”, they don’t address sources of research waste other than current publishing processes, such as waste resulting from avoidable design flaws, and incomplete or misleading reporting ( www.rewardalliance.net; Paul Glasziou and Iain Chalmers: Is 85% of health research really “wasted”?). The most clearcut example of these systemic problems is biased underreporting of research, with around half of all research going unpublished, implying $10s of billions wasted yearly.

Although underreporting can cause avoidable suffering and death, appeals to the biomedical research community beginning in the mid-1980s to deal with the problem had no discernible impact until this century ( https://clinicaltrials.gov/ct2/resources/trends). Change began when the International Committee of Medical Journal Editors required researchers to register controlled trials at inception (for example, at www.clinicaltrials.gov and www.isrctn.com), followed by the substantial community awareness of the scandal which followed publication of Ben Goldacre’s popular science book *Bad Pharma* and the launch of the AllTrials campaign ( www.alltrials.net). After these direct appeals to the public to call the research community to account, increasing numbers of research funders and regulators began to require researchers to behave more responsibly ( Moher *et al.*, 2015). But public recognition that self-regulation by researchers has failed has led to eroded trust in the biomedical research enterprise more generally.

As far as reporting of clinical trials is concerned, there has been slow progress towards the ideal of ‘threaded documents’, beginning with publication of the protocol, later publication of summary results, and going through to deposition of the final dataset ( Chalmers & Altman, 1999). But post-publication corrections, updates, simple linkages to similar studies and systematic reviews are also important for those trying to use, apply, or replicate studies.

We know remarkably little, formally, about why researchers do and don’t do the things that they do and don’t do. Some efforts to secure research funding to investigate why researchers don’t publish reports of their research have not been successful (Professor Mary Dixon-Woods, personal communication). If the attractive vision of a more efficient publishing model for the life sciences is to be promoted effectively, research is needed to find answers to the questions raised by Tracz and Lawrence themselves: why are researchers reluctant to post preprints, and will sufficient other researchers post useful and critical comments on them to make the effort worthwhile?

The current journal-based publication system is one of the important weak links leading to current waste from non-reporting and poor reporting of research. There is no doubt that new models are needed, but these publishing experiments also warrant formal evaluation and comparison to assess which of them delivers most effectively the advances needed. As far as clinical trials are concerned, methods and summary results are increasingly being posted on trials registries. More broadly, the National Institute for Health Research in England now requires publication of detailed reports for all the health technology assessments it supports. Among journals, PLOS One’s decisions to publish a report are now based on whether the research has used valid methods, regardless of the results.

Tracz and Lawrence and F1000 are well placed to foster the exploratory and evaluative research needed to inform future developments in science publishing, and we very much hope they will do so.
